# Provision of metadata of European agricultural long-term experiments through BonaRes and EJP SOIL collaboration

**DOI:** 10.1016/j.dib.2022.108226

**Published:** 2022-04-29

**Authors:** Cenk Donmez, Guillaume Blanchy, Nikolai Svoboda, Tommy D'Hose, Carsten Hoffmann, Wilfried Hierold, Katja Klumpp

**Affiliations:** a Leibniz Centre for Agricultural Landscape Research (ZALF), Muencheberg 15374, Germany; b Flanders Research Institute for Agriculture, Fisheries and Food (ILVO), Melle 9090, Belgium; c Cukurova University, Landscape Architecture Department, Remote Sensing and GIS Lab, 01330 Adana, Turkey; d Institut National de Recherche Pour l'agriculture, l'alimentation et l'environnement (INRAE), Grassland Ecosystem Research Unit, Clermont Ferrand 63000, France

**Keywords:** Long-term experiments, Europe, BonaRes, EJP SOIL, Agriculture, LTE, FAIR data principles

## Abstract

Agricultural Long-Term Experiments (LTEs) are crucial agricultural research infrastructures for monitoring the long term effects of management and environment on crop production and soil resources. We have compiled the meta-information of 616 LTEs from 30 different countries across Europe with a duration of typically 20 years, including clustered information of the European LTEs in different categories (management operations, land use, duration, status, etc.). It consists of the updated version of the dataset published by Grosse et al., (2020) but is extended by further LTE metadata, categories and research themes. Each set of metadata consists of up to 49 different attributes (categorical or numeric). Collected attributes were analyzed according to several research themes, including fertilization, crop rotation and tillage treatments. The collection of individual metadata was enlarged by the recent agreement between the BonaRes (www.bonares.de) and EJP SOIL (www.ejpsoil.eu) groups into the most comprehensive dataset in Europe, providing access to LTE and other, shorter running experiments. This dataset centralized past and existing information usually dispersed across several national actors. As such, it provides an extensive database that can be used by decision-makers, scientists, LTE owners and the public. The dataset can be updated in the future to foster networking and information exchange continuously.

## Specifications Table

**Table:** 

Subject	Agricultural Science
Specific subject area	Agronomy and Crop Science
Type of data	Figures, Tables
How data were acquired	The dataset was acquired by (i) a bibliographic review, (ii) personal communication with the LTE holders through the BonaRes factsheet [5] and (iii) adopting from the dataset (https://doi.org/10.20387/bonares-r56g-fgrw). The bibliographic review was done in scientific databases including Web of Science Core Collection (WoS) and SCOPUS through the search terms “long-term field experiment”, “long-term trials”, “agricultural experiment”. Additional data were provided by the partners personally contacted within the EJP SOIL consortium WP7 task 7.3. Gaps in the data sets were filled in a further literature review.
Data format	Primary data
Description of data collection	The BonaRes datasets (https://doi.org/10.20387/BonaRes-3tr6-mg8r, https://doi.org/10.20387/bonares-eff3-0mb4), collected from scientific journals, books, research notes and websites, were further improved and merged with a dataset from EJP SOIL (https://doi.org/10.20387/bonares-jwhj-z839). Personal communication with numerous LTE holders was set up by exchanging the factsheets information. Collected LTE metadata in the study were included and visualized through the “LTE overview map” (https://lte.bonares.de) developed and maintained within the BonaRes project.
Data source location	Institution: Leibniz Centre for Agricultural Landscape Research (ZALF) City/Town/Region: Müncheberg, Brandenburg Country: Germany
Data accessibility	**Repository name:** BonaRes Repository **Data identification number (DOI):**10.20387/bonares-40kc-2661 **Direct URL to data:**https://doi.org/10.20387/bonares-40kc-2661?

## Value of the Data

● The data comprises the most comprehensive metadata set of LTEs and other experiments in Europe through the combination of independent datasets of the BonaRes and EJP SOIL projects.
● The dataset primarily offers a wide selection of the LTE locations, research themes, and sources that the scientists and decision-makers can use for possible collaborations, even with LTE holders, intending to analyze the soil ecosystem services.
● It is a significant source of information about various LTEs for soil and environmental researchers, research institutions, local authorities, along with access to relevant information for future studies and cooperation.
● This LTE data collection established a new basic framework in this study to make data from different LTEs comparable and appraisable, even though there are no standard procedures for LTE set-up, treatment designs, minimum information and data management.

## 1 Data Description

Agricultural long-term experiments (LTEs) are experiments for monitoring plant and soil parameters such as yield under different environmental conditions and management practices. These experiments were set up on a large number of different soil textures and soil types to reveal the effects of management practices and environment on crop production and soil resources. They offer a unique look at the change in several soil properties over the long-term effect of investigated management practices. Under changing climate conditions, representative data and time series from LTEs will help decision-makers, environmental organizations, authorities, local governments and scientists to develop innovative (research) activities to mitigate climate deterioration on agricultural productivity

Since the LTEs are essential infrastructures for sustainable soil use and yield, information produced from this LTEs-based information attracted attention from many different research institutions and organizations. For instance, LTE-based data were acquired and managed by various national, international or global initiatives and networks, including GLTEN (Global long-term experiment network), ILTER (International long-term ecological research), IOSDV (International Organic Nitrogen Fertilization Experiments), NLFT (National Long-term Fertilization Trials, Hungary), RetiBio 2 (Italy), and the projects BonaRes and EJP SOIL. While each organization has its data management plan, the variety of actors makes that LTE-related information was dispersed across different databases, data holders and publications, which makes making it challenging to access and reuse the data.

Since 2016, an extensive metadata collection on LTEs has been compiled within the BonaRes project. The basis of this collection was the BonaRes factsheet and LTE metadata template for the structured collection of metadata and the BonaRes definition of which field experiments have the status long-term experiment. The BonaRes definition is as follows: "field experiments with a minimum duration of 20 years and a static design” [1]. EJP SOIL data has been built very closely on the Factsheet and metadata template developed and proven in BonaRes to collect metadata. However, there are differences in the definition. EJP definition: “running field experiments focusing on (sustainable) soil management and a minimum duration of 5-10 years with a statistically sound design, a control treatment and regular monitoring of crop/soil parameters. We have not filtered this dataset using the strict BonaRes defaults but also integrated LTE meeting the EJP definition. However, we offer simple filtering possibilities based on the duration of the experiment, and thus every user has the possibility to decide for himself. In this case, we found that expressing the differing LTE definitions of both BonaRes and EJP SOIL would be relevant for researchers to select the experiment duration used in their own research.

**Fig. 1:**
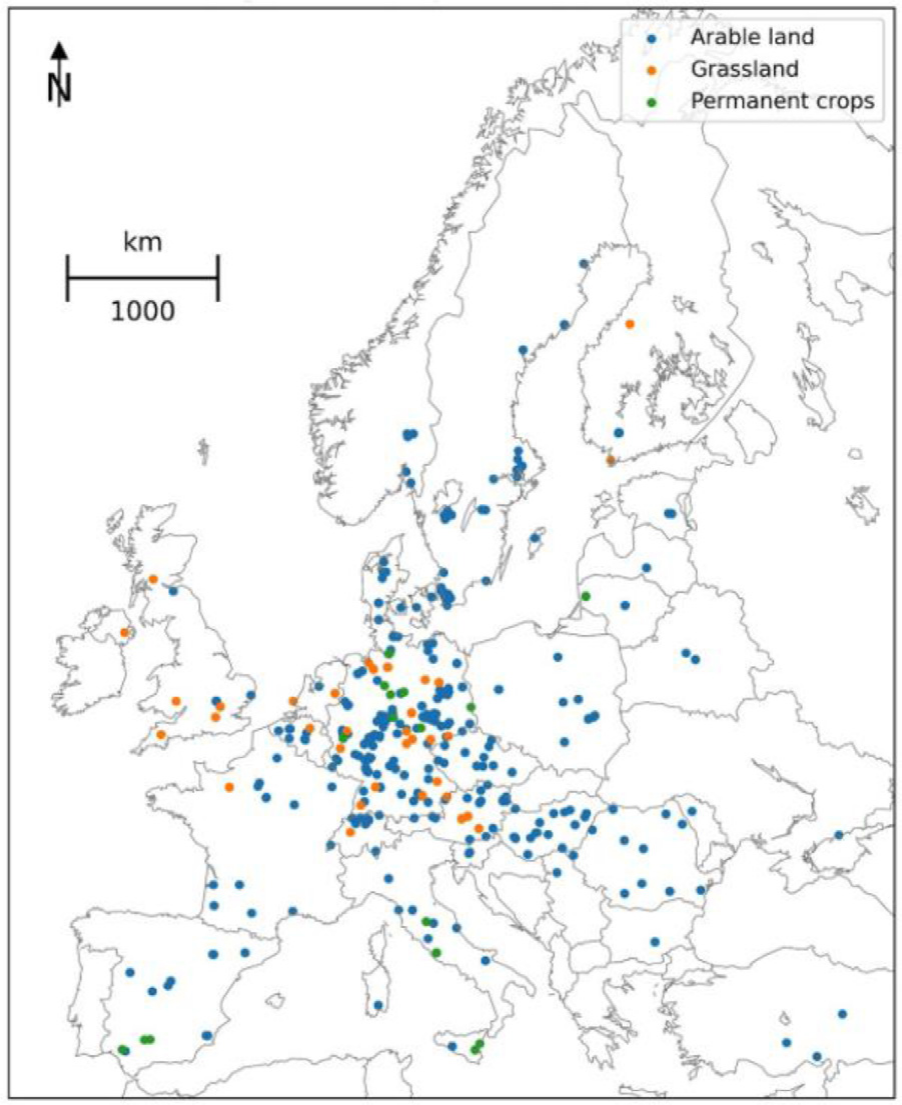
Location of LTEs in Europe provided in the study indicating the land use.

As the leading research initiatives, the BonaRes and EJP SOIL research groups have recently merged their LTE metadata databases of the European LTEs. Several LTE parameters and features for a time span of more than 150 yrs (1843–2022) were aggregated and published. The dataset comprises an updated number of the LTEs and revised research categories compared to the data published by Grosse et al. [1,3]. Recently the complete dataset consists of metadata for 616 LTEs from 30 countries in Europe (Fig. 1), that can be divided into five groups; (i) trial information (i.e. site, country, duration, status), (ii) land use (i.e., arable land, grassland), (iii) research theme, (iv) management operations (i.e. tillage, fertilization, crop rotation), and (v) basic soil parameters (i.e. types, texture).

Collected LTE data were merged and visualized through an online overview map (https://lte.bonares.de) running under the BonaRes Repository (https://datenzentrum.bonares.de). The overall dataset comprises the complete list of the LTEs and their attributes. The file includes 616 rows compatible with the listed LTEs and 49 columns of attributes in various categories. Besides the LTE-related attributes, the file consists of the cited literature and source information for each LTE. A comprehensive EXCEL sheet is provided as supplemental information along with the published dataset: https://doi.org/10.20387/bonares-40kc-2661.

The article displays two tables; (1) Table 1 includes the list, and description of the categories used for displaying into the Bonares LTE overview map, (2) Table 2 offers the ranges of values or the choices of the LTEs for each country and main categories.

**Table 1:** Description of the categories used for displaying into the Bonares LTE overview map.

Header	Description
index	index of the row
name	name of the experiment
site	name of the site
country	country
start_date	starting year
end_date	ending date, put 'ongoing' if still running
trial_duration	in years
trial_status	either 'Finished' or 'Ongoing'
trial_institution	institution managing the trial
landuse_type	Corine Land Cover types
research_theme	list of research themes investigated by the experiment
tillage_trial	yes if tillage is a factor investigated, else no
fertilization_trial	yes if fertilization is a factor investigated, else no
crop_rotation_trial	yes if crop rotation is a factor investigated else no
cover_crop_trial	yes if cover crop is a factor investigated else no
irrigation_trial	yes if irrigation is a factor investigated else no
pest_weed_trial	yes if pest/weed is a factor investigated else no
grazing_trial	yes if grazing is a factor investigated else no
other_trial	yes of other factors are investigated else no
holder_category	type of institution: university, non-university scientific institution, other institution
website	links redirecting to a website for more information about the experiments
networks	network name if the experiment is part of a network
research_parameters	list of variables measured in the experiments
farming_category	type of farming system (e.g. conventional, organic)
position_exactness	position of the coordinates compare to the field
size_hectares	size of the field
longitude	longitude as decimal degree
latitude	latitude as decimal degree
experimental_setup	setup of the experiment
tillage_levels	levels of tillage separated by |
fertilization_levels	levels of fertilization separated by |
crop_rotation_levels	levels of crop rotation separated by |
cover_crop_levels	levels of cover crops separated by |
irrigation_levels	levels of irrigation separated by |
pest_weed_levels	levels of pest/weed separated by |
grazing_levels	levels of grazing separated by |
randomization	randomization used
replication	number of replicates
number_plots	number of plots
size_plots	size of the plots
soil_group_wrb	soil group according to WRB
soil_type_other	soil type according to other classification
parental_material	parental material (rocks, alluvion, ...)
soil_info	other soil information
texture	soil texture according to USDA
texture_sand	sand content
texture_silt	silt content
texture_clay	clay content

bulk_density	bulk density
organic_carbon_prc	soil organic carbon
miscellaneous	miscellaneous
literature	paper reference separated by | if multiple
sources	other sources
agrovoc_keywords	keywords separated by

**Table 2:** Ranges of the LTEs for each country and main categories.

Column	Values	Unit	Number of gaps
country	Germany (299), Sweden (51), Austria (31), Great Britain (27), Hungary (23), France (18), Italy (18), Denmark (16), Switzerland (15), Spain (14), Romania (13), Poland (12), United Kingdom (12), Norway (11), Czech Republik (10), Belgium (10), Finland (6), Bulgaria (5), Ireland (4), Lithuania (4), Netherlands (3), Turkey (3), Slovenia (3), Belarus (2), Estonia (2), Serbia (1), Moldova (1), Latvia (1), Ukraine (1)	-	0
start_date	1843–2022	years	17
trial_duration	0–178	years	14
trial_status	Ongoing (508), Finished (82)	-	26
landuse_type	Arable land (505), Grassland (69), Permanent crops (19)	-	23
farming_category	Conventional (364), Organic (31), Conventional and organic (15)	-	206
tillage_trial	Yes (332), No (255)	-	29
fertilization_trial	No (448), Yes (139)	-	29
crop_rotation_trial	No (525), Yes (65)	-	26
cover_crop_trial	No (562), Yes (29)	-	25
irrigation_trial	No (584), Yes (5)	-	27
pest_weed_trial	No (521), Yes (13)	-	82
grazing_trial	No (531), Yes (5)	-	80
texture	Sandy loam (61), Silt loam (37), Loam (34), Clay loam (21), Silty clay loam (18), Sand (15), Clay (14), Loamy sand (13), Sandy clay loam (9), Others (9), Silty clay (6)	-	379

**Fig. 2:**
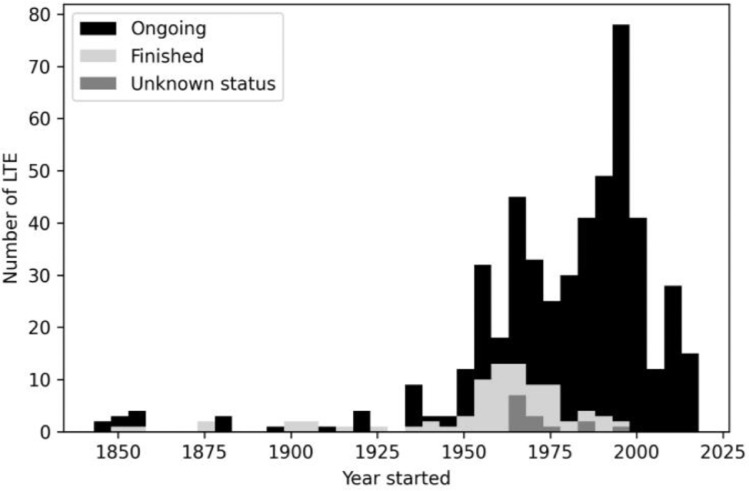
Histogram of starting date of European LTEs (*n =* 616).

While merging the BonaRes and EJP SOIL LTE sets, 19 doubles became visible. Fifteen of these LTEs are located in Germany, and one is from Switzerland, Belgium, Denmark, and Italy. Duplications of these LTEs were excluded from the dataset to prevent redundancies. Following the exclusion of duplicates, the final number of the LTEs were defined. The majority of the LTEs in Europe are located in Germany (*n* = 299), Sweden (*n =*  51), Austria (*n =* 31), Great Britain (*n =* 27). A considerable number of LTEs are also located in Mediterranean countries, including Italy (18), France (18), Spain (14) and Turkey (3), respectively. The world's oldest and still running LTE is the Broadbalk experiment (Rothamsted Research, Harpenden, UK), which started in 1843. Fig. 2 shows the number of LTE according to their starting date and current status.

**Fig. 3:**
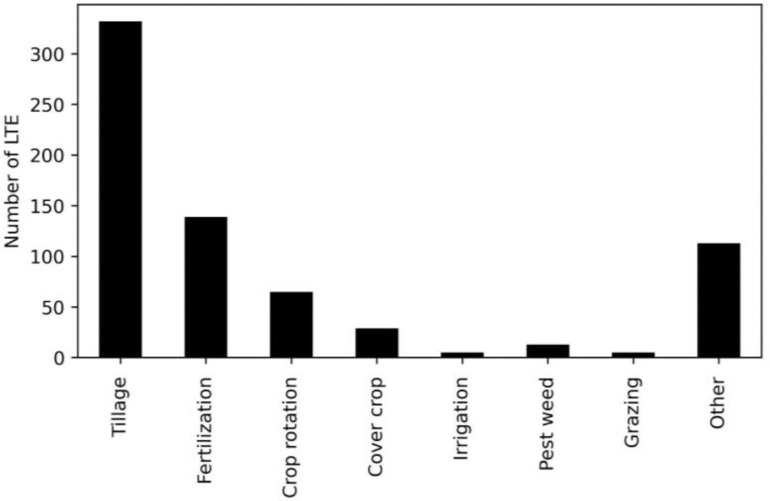
Bar plot showing the number of LTEs investigating a specific research theme (*n =* 616).

The majority of LTEs (*n =* 508) are still ongoing, where 82 LTEs have been finished (after running times of at least a typical 20 years). By far, most LTEs are categorized as crop rotation (65), fertilization (139) or tillage experiments (*n =* 322, Fig. 3).

The soil information of LTEs was presented in nine attributes representing the soil texture, types, bulk density, organic carbon and further soil information. These parameters were assigned for each LTE, if applicable. Where the data could not be inferred, we included “unknown” in the respective fields of the LTE tables.

## 2 Experimental Design, Materials and Methods

The study comprised the LTEs in Europe and merged the data of three datasets. Additional data were identified through a comprehensive bibliographic review and personal communication with numerous LTE holders. Articles from scientific journals, books, research notes and websites were selected addressing the research themes and parameters of the agricultural LTEs. At first, we conducted a systematic, keyword-based search in Web of Science (WoS) Core Collection (https://apps.webofknowledge.com/) and SCOPUS (https://www.scopus.com/) as well as the Google Scholar databases to create an overview of available LTE information. The bibliographic review was based on the English keywords “long-term field experiment”, “long-term experiment”, “long-term field trial”, and “long-term trial” in title, abstract or keywords [2]. However, as especially older publications are written in national journals; also national (German) keywords were applied, such as “Dauerfeldversuch”, “Dauerdüngungsversuch”, “Dauerversuch”, “Langzeitfeldversuch” and “Langzeitversuch” to precisely identify the LTEs in Germany where the majority of LTEs are located [2]. Articles representing LTE information were selected if they addressed at least the e.g. site, duration, land use and management operations. Thus, we recorded the metadata contents for each LTE as inputs listed for the dataset. Both English and German keywords were compatible with the multilingual thesaurus AGROVOC (FAO) that assisted in finding and linking LTEs in related topics. Data collection was carried out in 30 European countries and described based on AGROVOC keywords (https://agrovoc.fao.org/browse/agrovoc/en/).

Besides the bibliographic review, the metadata of the considerable number of LTEs was received by personal communication. Factsheets were forwarded to many LTE holders, and the filled versions were discussed via e-mails, phone calls, and online meetings. The LTE metadata was uploaded to the overview map once the information on the factsheet was agreed upon for the related experiment.

The data for the additional LTE were collected using the EJP SOIL metadata template developed from the keyword trees of the knowledge library and adapted for the need of the consortium, which consisted of a multi-sheet excel file with data validation and controlled vocabulary through drop-down lists. Copies of the template were automatically made using Google Drive API. Links to templates were distributed to national representatives within the EJP SOIL consortium then sent to the LTE owners. A script was implemented to download the templates and perform automatic checks. The automated checks included controlling the type of the values, if a value was inside the drop-down list or if it was a new choice added by the user. The automatic check review also verified the quality of the data; a.o. consistency of the index across the different tables of the template and their uniqueness. All templates that passed the automatic review were merged together to form the final database. The database was then exported to the format described above to be further processed and merged with datasets [1,^3^,4].

## Ethics Statements

None.

## CRediT authorship contribution statement

**Cenk Donmez:** Conceptualization, Methodology, Data curation, Writing – original draft, Supervision. **Guillaume Blanchy:** Conceptualization, Methodology, Data curation, Writing – original draft. **Nikolai Svoboda:** Conceptualization, Methodology, Writing – original draft, Supervision. **Tommy D'Hose:** Methodology, Data curation, Writing – original draft. **Carsten Hoffmann:** Methodology, Data curation, Writing – original draft. **Wilfried Hierold:** Methodology, Data curation, Writing – original draft. **Katja Klumpp:** Writing – original draft.

## Declaration of Competing Interest

The authors declare that they have no known competing financial interests or personal relationships that could have influenced the work reported in this paper.

## Data Availability

BonaRes_EJPSOIL_LTEmetadata (Original data) (BonaRes Repository)

## Appendix A. Dataset

The dataset described in this article is available online at the BonaRes Repository at Leibniz Centre for Agricultural Landscape Research (ZALF), Germany (https://doi.org/10.20387/bonares-40kc-2661). Download is not restricted, and usage is regulated by CC BY 2.0 (https://creativecommons.org/licenses/by/2.0/) license.
